# Customized positioning of the glenoid component in reverse shoulder arthroplasty: a new computer aided design methodology

**DOI:** 10.1007/s00264-026-06748-9

**Published:** 2026-02-07

**Authors:** Antonino Cirello, Tommaso Ingrassia, Giuseppe Rovere, Lorenzo Nalbone, Lawrence Camarda, Igor Agostino Mirulla, Vincenzo Nigrelli, Vito Ricotta, Micol Tantillo

**Affiliations:** 1 Department of Engineering, Università degli Studi di Palermo, Viale delle Scienze Ed. 8, Palermo, Italy; 2 Tor Vergata University Hospital (Policlinico Tor Vergata), Roma, Italy; 3 University of Rome Tor Vergata, Rome, Italy; 4 Department of Orthopaedics and Traumatology, Fondazione Policlinico Universitario A. Gemelli IRCCS - Università Cattolica del Sacro Cuore, Largo Agostino Gemelli, 8, 00168 Roma, Italy; 5 Department of Precision Medicine in Medical, Surgical and Critical Care (Me.Pre.C.C.), University of Palermo, Palermo, Italy

**Keywords:** Reverse shoulder arthroplasty, CAD-based planning, Glenoid positioning, Range of motion, Joint stability, Patient-specific modeling

## Abstract

**Purpose:**

Reverse Shoulder Arthroplasty (RSA) is widely used to treat shoulder joint pathologies. However, this procedure may result in reduced range of motion (ROM), scapular notching, and prosthetic instability. These complications vary among patients, highlighting the need for individualized preoperative planning. This study introduces a novel parametric methodology to determine optimal glenoid component positioning by evaluating ROM, instability ratio, and the percentage of bone resected.

**Method:**

The proposed approach was applied to four patient models treated with two prosthetic designs. The methodology consists of four steps within a patient-specific parametric tool: 3D anatomical reconstruction, virtual surgical planning, biomechanical and geometric evaluation, and identification of optimal configurations. Fifteen glenoid component orientations were generated by varying tilt angles. The best configurations were identified based on ROM and instability assessments, while bone resection volume was calculated as an additional parameter.

**Results:**

Maximum values of abduction–adduction, internal rotation, and external rotation were 87.23°, 90°, and 70.59°, respectively, although not achieved in a single configuration. Instability ratios ranged from 0.23 to 0.62. Bone resection varied between 0.4% and 5.5%, depending on the configuration.

**Conclusions:**

This methodology provides a patient-specific framework to support preoperative planning in RSA. By combining ROM analysis, instability assessment, and bone preservation, the approach enables the identification of glenoid component orientations that improve mobility while minimizing instability risk and surgical invasiveness.

## Introduction

Reverse Shoulder Arthroplasty (RSA) is a well-established surgical procedure for the treatment of severe rotator cuff arthropathy and other complex shoulder pathologies. While RSA provides clear biomechanical advantages—such as enhanced deltoid efficiency and improved upper limb mobility—it is also associated with complications. These include reduced range of motion (ROM), prosthetic instability, and scapular notching. These complications are clinically significant, occurring in 9–40% of cases, often necessitating revision surgery (10–25% rates) and severely impairing patients' quality of life through persistent pain, limited daily activities (e.g., dressing, overhead reaching), reduced independence, and lower scores on functional scales like *Constant-Murley* or *Disabilities of the Arm, Shoulder and Hand* (DASH) [1, 2]. Improved preoperative assessment of factors influencing complications associated with reverse shoulder arthroplasty is important for patient counseling and improving outcomes, given their impact on postoperative recovery and long-term function [3]. Previous studies have shown that implant positioning, soft tissue tension, muscle function, and preoperative planning are key factors influencing RSA outcomes [4–9].

Among these specific complications, scapular notching (prevalence 20–50%) leads to progressive glenoid bone loss, polyethylene wear, and humeral component loosening over time, compromising long-term implant survival and function. Prosthetic instability (3–13%) similarly results in recurrent dislocations, chronic pain, and early failure, with revision rates up to 30% in affected cases [10, 11].

The positioning of the glenoid component plays a critical role in determining RSA outcomes [7–9, 12] Currently performed manually, this step plays a central role in preventing adverse events such as mechanical impingement and glenoid migration, both of which can compromise joint stability and ROM [12, 13].

Glenoid migration, although relatively rare (1.7–3.5%), is frequently attributed to inappropriate implant sizing or malpositioning [14].

Conversely, mechanical impingement due to glenoid misalignment is common and typically occurs between the humeral component and the scapula, or between the humerus and the acromion [15].

Another frequent complication is prosthetic instability, which results from abnormal movement or dislocation of the implant. Instability may be caused by mechanical imbalance, inadequate soft tissue tension, muscle dysfunction, or incorrect implant placement [3].

In this context, computer-assisted preoperative planning has emerged as a valuable tool to improve surgical accuracy. By integrating patient-specific anatomical data, virtual planning enhances the surgeon’s ability to select the optimal implant type, size, and orientation, supporting a more personalized approach to RSA [3, 16–22].

Nevertheless, current computer-assisted methods are limited, as they typically assess only a few well-known ROM conditions that may lead to impingement [23, 24].

The present study proposes a parametric methodology for patient-specific preoperative planning in RSA. The primary aim is to evaluate glenohumeral (GH) ROM and prosthetic instability across different glenoid tilt configurations. Fifteen orientations were simulated by varying inferior, anterior, and posterior tilt angles of the glenoid cutting plane. While three of these correspond to standard clinical practice, twelve additional configurations were introduced as potential alternatives. Finally, for each optimal configuration, the percentage of bone resection required was also calculated, providing an additional parameter to support surgical decision-making. This approach holds potential clinical promise by enabling surgeons to select glenoid tilts that may maximize ROM, minimize instability risk, and optimize bone preservation, which may contribute to reduced complication rates, improved long-term outcomes, and enhanced patient satisfaction and function.

## Methods and applications

### Study design and workflow

**Fig. 1:**
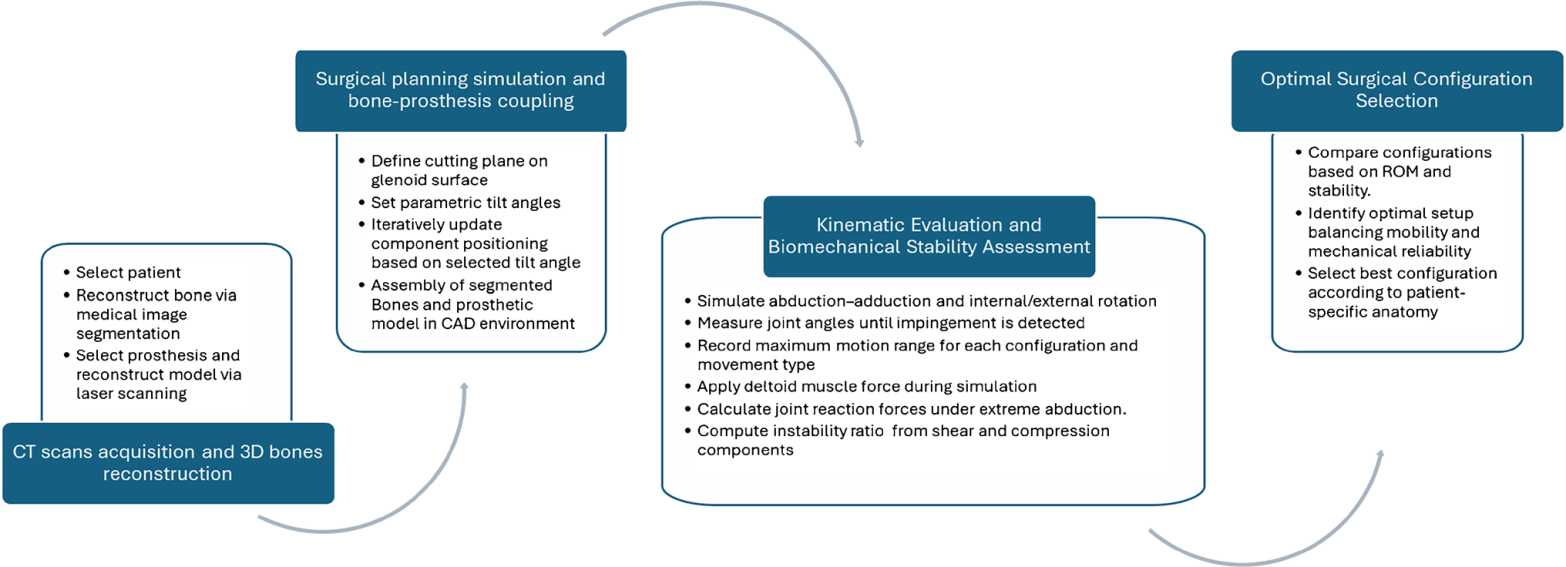
Flow chart of the methodology for preoperative planning in reverse shoulder arthroplasty

This study applied a patient-specific parametric methodology for preoperative planning in RSA. The workflow, summarized in Fig. 1, consists of four sequential steps, each critical for ensuring anatomical accuracy, implant optimization, and clinical translation:

- CT data acquisition and 3D bone reconstruction, providing patient-specific geometry, essential for personalized implant positioning.
- Virtual surgical planning and bone–prosthesis coupling, enabling precise glenoid tilt simulation to avoid malpositioning and/or severe surgical problems (i.e. over reaming).
- Geometric evaluation of ROM, identifying impingement-free configurations maximizing functional outcomes and subsequent Biomechanical Instability Assessment, quantifying instability risk under loading conditions.
- Bone resection analysis, balancing stability gains against bone preservation for long-term implant survival

**Fig. 2:**
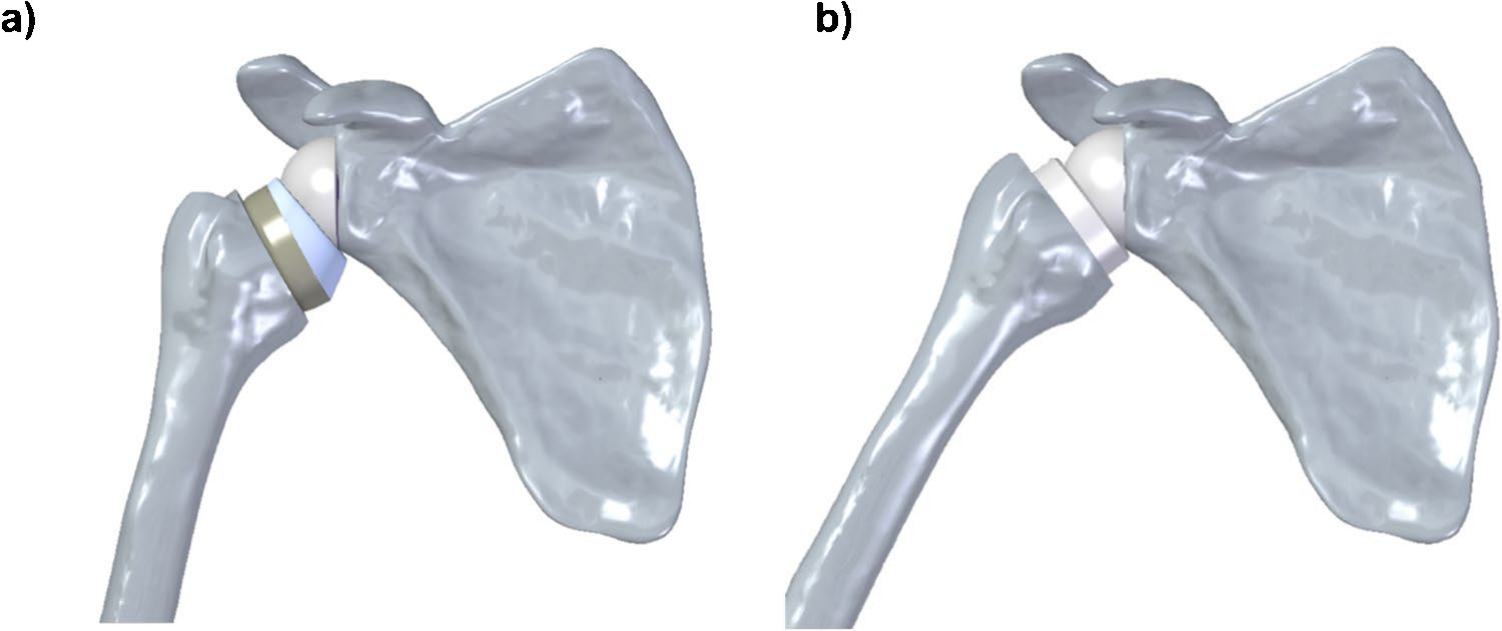
*Onlay* design (**a**); *Inlay* design (**b**)

The methodology was tested in four clinical cases using two different prosthetic designs: an *Inlay* straight stem [25] and an *Onlay* curved stem [26] (Fig. 2).

#### CT data acquisition and 3D reconstruction

CT images of the shoulder joint were acquired using a Siemens Sensation 16 scanner (pixel spacing 0.496 × 0.496 mm, resolution 512 × 512, slice thickness 1 mm). From these datasets, 3D models of the humerus and scapula were reconstructed using 3D Slicer 5.0. Segmentation was performed semi-automatically by applying a predefined grayscale threshold, followed by manual refinement to remove artifacts and smooth bone contours. High-resolution CT reconstruction ensures sub-millimeter accuracy in glenoid morphology reconstruction, which is critical for reproducible and accurate cutting plane definition. All patient data were anonymized to ensure privacy.

#### Virtual surgical planning and bone–prosthesis coupling

A reference configuration (labeled A, Table 1) was created according to standard surgical guidelines. [26] The scapular coronal plane was defined using three anatomical landmarks: the center of the glenoid fossa, the most medial point of the scapular spine, and the most distal point of the inferior angle. A cutting plane perpendicular to this reference was established for the initial configuration [21].

**Table 1:** Tilt configurations of the glenoid component

Angle for creation of cutting planes on the glenoid cavity			
Configuration	Glenoid inferior tilt	Glenoid posterior tilt	Glenoid anteriortilt
A	0°	0°	0°
B	0°	0°	2°
C	0°	0°	3°
D	0°	5°	0°
E	0°	10°	0°
F	5°	0°	0°
G	5°	10°	0°
H	5°	5°	0°
I	5°	0°	2°
J	5°	0°	3°
K	10°	0°	0°
L	10°	0°	2°
M	10°	0°	3°
N	10°	10°	0°
O	10°	5°	0°

**Fig. 3:**
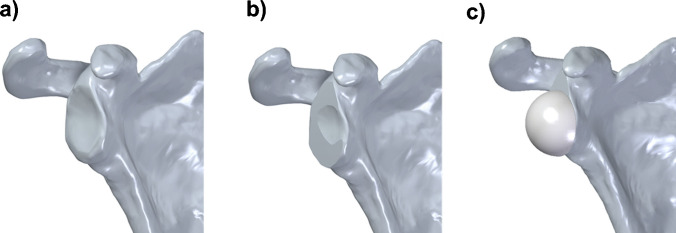
Glenoid preparation and component positioning: native glenoid surface (**a**); planar surface obtained through reaming, to allow proper seating of the glenoid component (**b**); final fixation of the glenosphere on the reamed glenoid (**c**)

In a Solid Edge 2021 virtual environment, glenoid reaming was simulated to create a planar surface ensuring proper seating and fixation of the baseplate (Fig. 3a). The choice of the software is not critical, and potentially, other 3D parametric modelling software with a motion analysis module could be used. Additional procedural steps were incorporated into the surgical procedure, including humeral head resection and canal preparation. The alignment of the metaphyseal axis was taken to ensure consistent prosthesis alignment. The depth of the cut (Fig. 3b) was optimized to ensure stable fixation without over-reaming. [12] Final fixation of the glenosphere was then simulated (Fig. 3c).

**Fig. 4:**
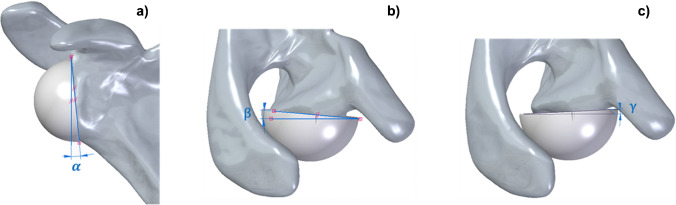
Views of the glenoid component illustrating the angle parameters used to generate different glenoid cutting configurations

To explore alternative orientations, the inclination of the glenoid cutting plane was systematically modified. The inferior tilt was set at 0°, 5°, and 10°; posterior tilt at 0°, 5°, and 10°; and anterior tilt at 0°, 2°, and 3°. These parameters generated 15 unique configurations (Table 1), including three commonly used in clinical practice (A, F, K). Configurations B–E explored isolated anterior or posterior tilts, while G–O combined inferior tilt with either anterior or posterior tilt. Figure 4 illustrates the definition of the α (inferior), β (anterior), and γ (posterior) tilt angles relative to scapular reference planes.

The objective of parametric variation is to systematically explores the design space beyond standard configurations, identifying patient-specific alternative solutions that are not typically considered during manual preoperative planning [27–30].

#### Geometric evaluation of ROM

Glenohumeral ROM was assessed for three movements: [31]

**Fig. 5:**
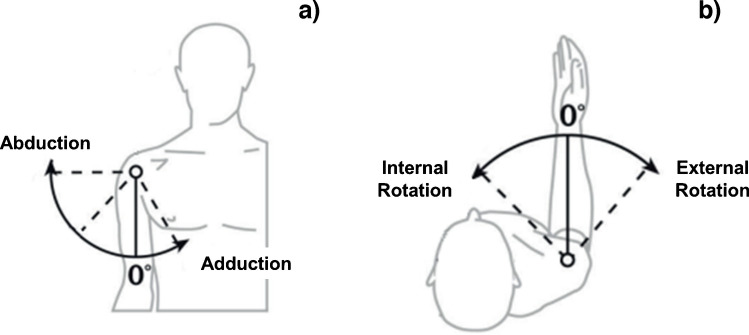
Shoulder joint range of motion: Abduction and adduction in the frontal plane (**a**); Internal and external rotation with the elbow flexed at 90° (**b**)

- **Abduction–Adduction (Ab–Ad)**: angle between the humeral axis and the body midline in the frontal plane (Fig. 5a) [27].
- **Internal Rotation (IR) and External Rotation (ER)**: medial and lateral rotations of the forearm around the humeral axis with the elbow flexed at 90° (Fig. 5b).

**Fig. 6:**
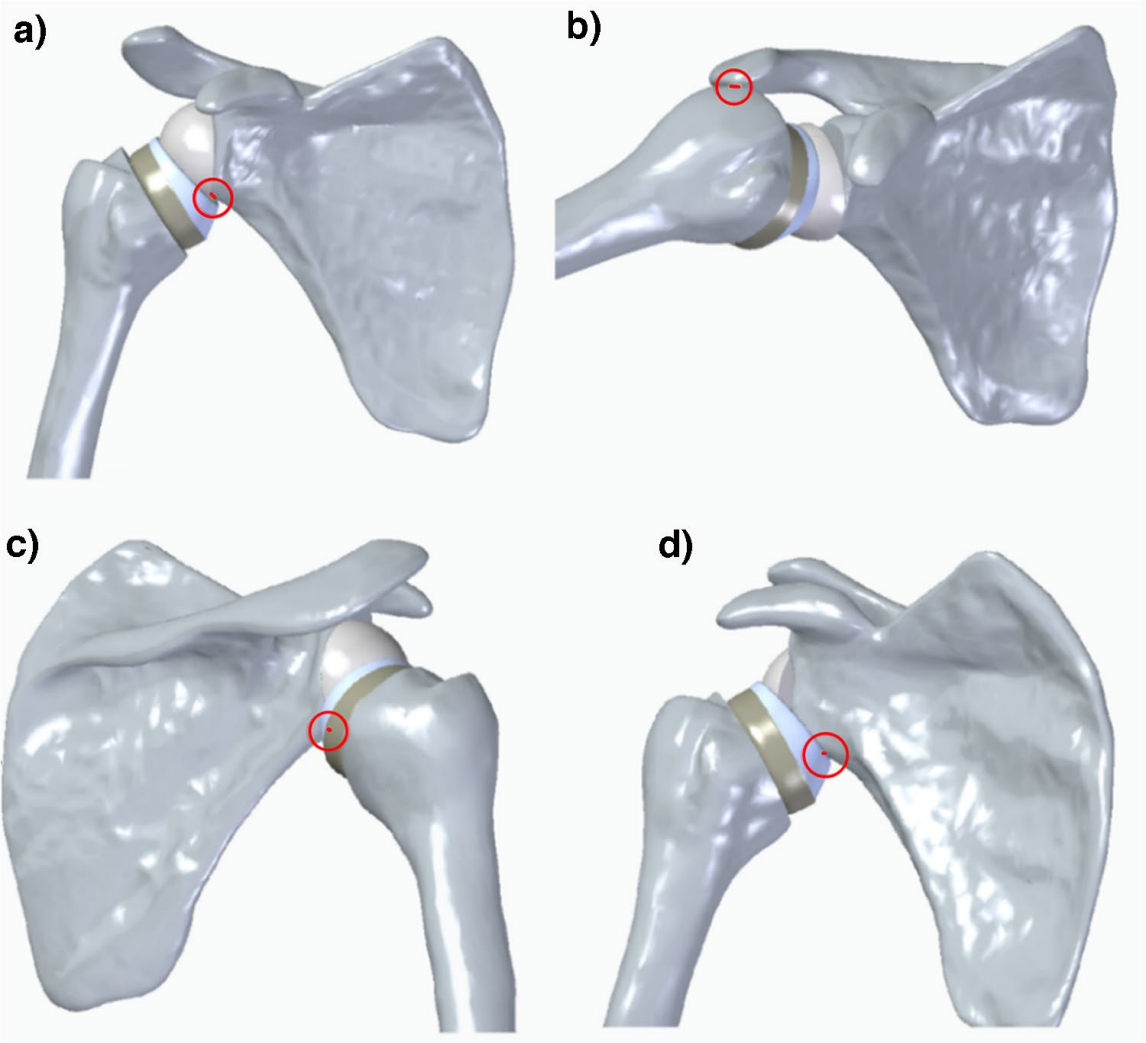
Impingement conditions: **a**) between the polyethylene and the posterior glenoid; **b**) between the greater tuberosity and the acromion; **c**) occurring during external rotation of the arm; **d**) occurring during internal rotation of the arm

Movements were simulated until impingement occurred. Upper impingement was defined as humerus-to-acromion contact, while lower impingement was defined as humerus- or polyethylene-to-scapula contact (Fig. 6).

#### Biomechanical instability Assessment

Joint stability was evaluated using a numerical approach within Solid Edge. Each configuration was analyzed at maximum abduction (0–90° range), where deltoid force peaks and the rotator cuff is inactive. [32]

**Fig. 7:**
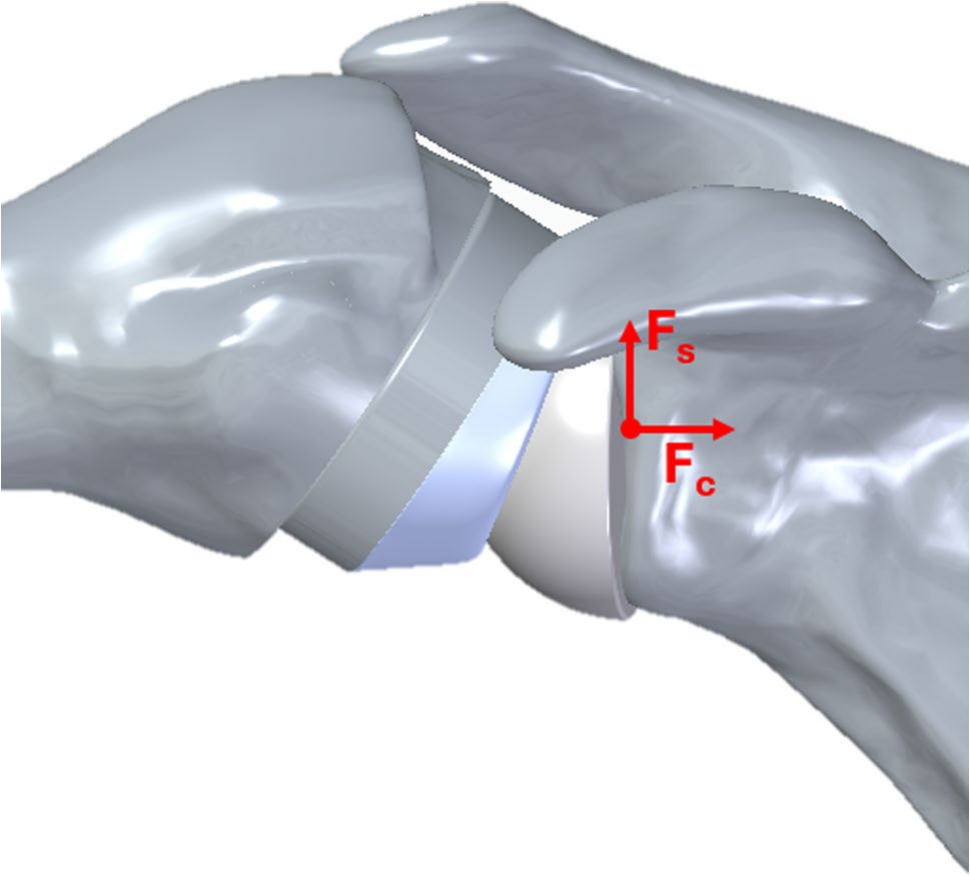
Joint reaction force acting on the center of rotation of the glenosphere: shear (Fs) and compressive (Fc) components

Deltoid force (240 N, representing maximum isometric contraction) was applied based on anatomical insertion points, together with the weight of the upper limb. Instability risk was quantified as the ratio between shear force (Fs), parallel to the glenoid plane, and compressive force (Fc), perpendicular to the plane, acting at the glenosphere’s center of rotation (Fig. 7) [13, 33]. A higher Fs/Fc ratio indicates greater instability risk.

#### Bone resection analysis

For configurations achieving the best ROM and stability outcomes, the percentage of bone volume removed during glenoid preparation was quantified by comparing pre- and post-reaming models. This metric was included as an additional factor for surgical decision-making.

## Results

In the following, the outcomes of the performed geometric positioning simulations for each patient and implant configuration were reported. In particular, the results concerning the GH ROM have been included in Table 2, 3, 4, 5, and those regarding the instability ratio in Table 6.

**Table 2:** Patient 1's ROM

	*Onlay* design			*Inlay* design		
Configuration	Ab-Ad [°]	IR [°]	ER [°]	Ab-Ad [°]	IR [°]	ER [°]
A	60,07	49,49	13,08	53,04	13,70	11,78
B	58,83	48,73	10,52	55,72	11,09	4,25
C	58,28	45,08	8,36	66,59	11,07	3,24
D	62,77	58,28	10,15	62,25	8,20	5,32
E	**73,12**	68,3	4,6	62,38	26,69	5,06
F	63,35	73,34	21,02	63,47	42,20	7,97
G	64,06	55,97	22,89	54,57	26,42	14,97
H	64,74	90	10,24	57,70	66,72	14,25
I	62,78	69,86	16,96	63,48	52,74	9,42
J	61,46	71,31	17,32	62,10	43,00	12,62
K	65,58	90	26,75	**66,90**	**68,24**	**36,29**
L	**72,34**	**90**	**35,52**	67,46	70,49	31,79
M	70,22	90	33,65	61,48	**90**	20,98
N	**64,74**	**90**	**37,74**	64,96	72,93	25,58
O	70,37	90	33,55	**67,58**	**80,46**	**32,51**

Best configurations were reported bolded and underlined

**Table 3:** Patient 2's ROM

	*Onlay* design			*Inlay* design		
Configuration	Ab-Ad [°]	IR [°]	ER [°]	Ab-Ad [°]	IR [°]	ER [°]
A	**87,23**	**78,18**	**33,32**	67,39	50,73	9,42
B	83,35	77,42	30,00	69,26	63,91	6,63
C	83,35	77,75	30,13	68,38	39,86	6,63
D	84,43	73,75	**35,48**	67,39	20,87	5,94
E	82,76	73,66	29,35	67,82	46,41	6,36
F	79,43	**90**	27,20	67,31	68,72	26,31
G	69,62	71,66	35,03	66,89	80,14	25,83
H	78,92	80,25	30,80	64,86	58,83	23,95
I	**83,54**	**90**	**33,23**	67,33	57,60	26,42
J	**83,57**	**90**	**32,35**	66,89	74,21	21,09
K	75,93	**90**	26,83	**76,25**	**90**	**37,50**
L	81,20	**90**	35,15	**73,17**	**90**	**34,15**
M	80,03	**90**	32,02	73,17	90	28,42
N	72,09	55,61	29,58	62,94	45,11	37,82
O	73,26	**90**	21,74	68,76	49,88	**42,93**

Best configurations were bolded and underlined

**Table 4:** Patient 3's ROM

	*Onlay* design			*Inlay* design		
Configuration	Ab-Ad [°]	IR [°]	ER [°]	Ab-Ad [°]	IR [°]	ER [°]
A	78,23	65,56	43,75	65,92	20,59	36,91
B	76,08	68,65	45,13	67,08	21,52	26,33
C	76,07	68,25	41,16	68,87	21,45	1,27
D	**81,50**	82,94	36,96	64,22	16,89	19,67
E	74,99	81,03	34,29	69,31	47,87	20,26
F	76,45	86,47	42,61	75,93	48,99	39,84
G	72,43	81,43	29,75	64,70	19,73	33,86
H	**78,79**	**90**	**37,06**	**74,04**	**79,12**	**32,23**
I	76,96	85,25	44,62	**76,59**	60,63	43,87
J	75,73	83,66	45,63	73,58	53,53	40,29
K	71,90	86,51	34,54	73,18	38,52	47,33
L	**76,62**	**88,73**	**53,19**	66,63	44,35	37,12
M	**75,39**	**88,42**	**52,69**	66,63	46,85	35,96
N	67,14	68,77	**70,59**	60,44	43,35	55,11
O	**76,51**	**87,78**	**70,59**	**67,31**	**78,28**	**49,82**

Best configurations were bolded and underlined

**Table 5:** Patient 4's ROM

	*Onlay* design			*Inlay* design		
Configuration	Ab-Ad [°]	IR [°]	ER [°]	Ab-Ad [°]	IR [°]	ER [°]
A	67.37	89.36	26.64	58,38	52,31	10,19
B	68.93	89.68	26.32	59,78	59,36	7,98
C	68.94	89.04	24.55	57,17	51,47	1,60
D	71.03	90	21.53	68,68	54,14	41,14
E	76.76	90	24.55	65,12	25,12	21,82
F	68.14	90	24.27	64,70	22,58	13,45
G	78.84	90	39.79	71,57	88,99	32,57
H	76.76	90	39.40	73,92	88,99	32,57
I	74.11	90	36.83	69,63	90	17,11
J	75.02	90	39.72	67,33	90	16,19
K	73.59	90	31.71	70,50	23,28	**54,38**
L	74.11	90	42.17	**79,30**	**90**	**47,52**
M	**75.91**	**90**	**44.65**	77,53	90	36,66
N	72.91	90	24.45	69,72	86,32	41,29
O	**79.85**	**90**	**47.83**	**78,47**	**90**	**44,13**

Best configurations were bolded and underlined

**Table 6:** Instability ratio of selected best configurations

	*Onlay* design		*Inlay* design	
	Configuration	Instability ratio	Configuration	Instability ratio
Patient 1	L	0.23	K	0.46
	N	0.28	O	0.44
Patient 2	A	0.47	K	0.48
	I	0.68	L	0.52
	J	0.71		
Patient 3	O	0.35	O	0.47
	L	0.36	H	0.48
	H	0.47		
	M	0.38		
Patient 4	O	0.52	O	0.53
	M	0.54	L	0.56

### GH ROM assessment

The GH’s ROM analysis conducted across all patients and configurations (A–O) has highlighted a pronounced variability depending on the implant positioning and design.

For the patient 1, using *Onlay* implant design, the highest Ab-Ad rotation value was observed in configuration E (73.12°), while the lowest occurred in C (58.28°). IR has reached the maximum set value (90°) in six configurations (H, K, L, M, N, O), with a lower one (45.08°) in configuration C. ER ranged from a maximum value (37.74°) in configuration N to a minimum one of 4.6° in E.

Using an *Inlay* implant design, the highest Ab-Ad rotation value ranged from 53.04° (A) to 67.58° (O). The maximum IR value have been reached in M (90°), while the minimum one in D (8.20°). ER ranged from 3.24° (C) to 36.29° (K). All values have been reported in Table 2.

For patient 2, with an *Onlay* implant, the Ab-Ad rotation peaked at 87.23° (A), with the lowest value at 69.62° (G). IR reached 90° in 10 out of 15 configurations, with a minimum value of 55.61° (N). ER ranged from a minimum of 21.74° (O) to a maximum of 35.15° (L).

Using an *Inlay* design, the Ab-Ad ranged from 62.94° (N) to 76.25° (K). The IR peaked at 90° in 6 configurations (F, I, J, K, L, M, and O), with the lowest value in configuration D (20.87°). ER showed the widest variability, from 6.36° (E) to 42.93° (O). All values have been reported in Table 3.

For patient 3, equipped with an *Onlay* implant, the maximum Ab-Ad value was recorded in configuration D (81.50°), and the minimum in configuration N (67.14°). The allowed IR has reached very high values, with 4 configurations (H, L, M, and O) close to the maximum allowed rotation of 90°, and a minimum value of 65.56° in configuration A. ER ranged from 29.75° (C) to 70.59° (O).

Instead, Ab-Ad values ranged from 60.44° (N) to 76.59° (I), the IR from 79.12° (H) to 16.89° (D), and the ER from 55.11° (N) to 1.27° (C), by considering an *Inlay* implant design. All values have been reported in Table 4.

Lastly, a range values of Ab-Ad from 67.37° (A) to 79.85° (O), of IR almost close to 90° in most configurations, and ER from 21.53° (D) to 47.83° (O) have been obtained for patient 4 equipped with an *Onlay* implant design.

Using an *Inlay* design, the Ab-Ad values varied between 57.17° (C) and 79.30° (L), the IR stayed consistently ranged from 22.58° (F) to 90° (I, J, L, M, and O), and the ER showed a wide variability, with the lowest value of 1.60° (C) and the highest of 54.38° (K).

### Instability ratio

Instability analysis further differentiated the best-performing configurations (Table 6).

- Onlay design: ratios ranged from 0.23 (Patient 1, L) to 0.71 (Patient 2, J).
- Inlay design: ratios were consistently higher, between 0.44 (Patient 1, O) and 0.56 (Patient 4, L).

On average, the Onlay design provided lower instability risks compared to the Inlay design.

### Bone resection

The percentage of bone volume removed for optimal configurations is reported in Table 7.

- Minimum resection values were: 2.62% (Patient 1, K), 1.59% (Patient 2, I), 2.51% (Patient 3, H), and 1.99% (Patient 4, L).
- Maximum resection values were: 5.50% (Patient 1, N), 2.17% (Patient 2, L), 3.02% (Patient 3, O), and 2.39% (Patient 4, O).

**Table 7:** Percentage of bone tissue removed during cutting procedure in best configurations

		Bone Volume [mm^3]	Bone removed [%]
Pt1	Healthy	86105	
	A	85665	0.51
	L	83661	**2.84**
	K	83851	2.62
	N	81373	5.50
	O	82737	3.91
Pt2	Healthy	105625	
	A	104963	0.63
	I	103940	1.59
	J	103725	1.80
	K	103609	1.91
	L	103329	2.17
Pt3	Healthy	87711	
	A	87165	0.62
	H	85506	2.51
	L	85339	2.70
	M	85098	2.98
	O	85063	3.02
Pt4	Healthy	90029	
	A	89672	0.40
	L	88240	1.99
	M	88107	2.13
	O	87874	2.39

These findings indicate that bone preservation strongly depends on configuration, with several promising orientations minimizing both resection and instability risk.

Table 8 reports and compares the aggregate performance (mean ± SD) of the implant design with clinically significant reference ranges commonly reported in the literature.

**Table 8:** Clinical correlation of aggregate performance (mean values of Ab-Ad, IR, ER, Instability ratio, and Bone resection percentage) by implant design

	Onlay	Inlay	Clinical Significance
Ab-Ad (°)	74.2 ± 8.1	66.8 ± 7.4	> 70° = functional overhead reach [34]
IR (°)	82.5 ± 11.2	52.3 ± 25.4	> 70° = full reach behind back; < 50° = dressing limitation [35]
ER (°)	32.1 ± 12.3	24.6 ± 15.2	> 30° = perineal hygiene, desk work[36]
Instability ratio	0.42 ± 0.15	0.50 ± 0.12	< 0.5 = stable (< 5% dislocation)[19]
Bone resection (%)	2.3 ± 1.2	2.8 ± 1.4	< 5% = excellent preservation[36]

## Discussion

The results of this study demonstrate that optimizing glenosphere positioning can substantially improve glenohumeral (GH) mobility after RSA. Gains in abduction–adduction and rotational movements ranged from approximately 20% to 80%, depending on patient-specific anatomy and implant configuration.

Importantly, none of the configurations currently used in surgical practice (A, F, K) provided the best outcomes across all three ROM directions simultaneously. This finding highlights the limitations of standard surgical orientations and underscores the need for individualized preoperative planning.

The interactive tool developed in this study identified alternative configurations that offered a more favourable balance among Ab–Ad, IR, and ER. For example, configurations K, L, N, and O were most promising for Patient 1; A, I, J, K, and L for Patient 2; H, L, M, and O for Patient 3; and L, M, and O for Patient 4. These results support the clinical value of using patient-specific planning tools to expand beyond conventional orientations and explore individualized solutions.

However, the analysis also revealed trade-offs between improved ROM and joint stability. High Fs/Fc ratios (> 0.7) indicate increased dislocation risk (15–25%) and revision rates (25–30%), guiding surgeons to exclude unstable configurations despite ROM gains and prioritize stability-first planning during intraoperative adjustments [19]. In several cases, configurations associated with greater mobility were also linked to higher shear forces and increased instability risk. Therefore, ROM optimization alone cannot be considered sufficient to ensure favourable postoperative outcomes.

The bone resection analysis provides surgeons a quantitative metric to minimize complications: < 5% resection preserves > 95% glenoid stock, reducing loosening/notching risk (20–50% prevalence) and facilitating future revisions (10–25% RSA fate). Composite ranking (ROM + stability + resection) identifies optimal configurations for each patient anatomy [36].

The inclusion of instability ratios and bone resection percentages in the evaluation provides a more comprehensive perspective, allowing surgeons to identify configurations that balance mobility, stability, and bone preservation.

Overall, the Onlay design demonstrated lower instability risks compared with the Inlay design, particularly in patients 1 and 2, while Patient 3 exhibited greater variability across configurations. For Patient 4, the different configurations yielded similar results in terms of both instability and bone preservation, suggesting a broader range of acceptable surgical options.

These findings align with previous studies showing that implant design and positioning critically influence RSA biomechanics. [9, 19, 20, 37] Furthermore, they reinforce the concept that no universal “best” configuration exists. Instead, outcomes depend on the interplay between prosthesis design and individual scapular and humeral anatomy. [12, 13, 20, 38–40] In this context, the proposed parametric methodology represents a step toward more reliable and patient-specific surgical planning, enabling surgeons to achieve a compromise between mobility, stability, and surgical invasiveness.

### Limitations

First, the simulations focused exclusively on glenohumeral joint motion, without considering the contribution of the scapulothoracic articulation. Although this approach reduces the physiological accuracy of full shoulder kinematics, it was intentionally adopted to define impingement as the limiting condition of GH motion within a controlled and reproducible framework, minimizing variability from thoracoscapular dynamics.

Second, the biomechanical analysis considered only the deltoid muscle, while disregarding the active contribution of other shoulder muscles. This choice was made to isolate prosthesis-driven kinematics and evaluate implant stability under simplified loading conditions, in line with similar computational studies. [7, 33]

Finally, the study focused exclusively on the biomechanical implications of glenoid tilt variations, without addressing the surgical feasibility or reproducibility of each configuration. Future research should investigate how these orientations can be reliably transferred into intraoperative practice and validated through clinical outcomes.

## Conclusion

This study presented a parametric, CAD-based methodology for optimizing glenoid component positioning in reverse shoulder arthroplasty. By integrating patient-specific anatomical models with dynamic simulations, the method enabled systematic evaluation of implant configurations in terms of range of motion (ROM), joint stability, and bone preservation.

The results demonstrated substantial variability among patients and configurations. Notably, no single orientation simultaneously maximized all three ROM directions. In several cases, configurations providing enhanced ROM were also associated with increased shear forces and higher instability risk. These findings confirm that achieving optimal outcomes requires balancing mobility, stability, and surgical invasiveness on a patient-specific basis.

The proposed methodology offers a structured and interactive framework for preoperative planning, supporting surgeons in exploring alternatives beyond standard surgical practice. Rather than identifying a universal “best” configuration, the tool highlights a set of patient-tailored options, guiding surgical decision-making toward biomechanically favourable solutions.

Future developments should focus on refining the computational framework, ensuring reproducibility in intraoperative settings, and validating the approach through prospective clinical studies.

## Data Availability

No datasets were generated or analysed during the current study.
